# Progress of cGAS-STING signaling pathway-based modulation of immune response by traditional Chinese medicine in clinical diseases

**DOI:** 10.3389/fimmu.2024.1510628

**Published:** 2024-12-16

**Authors:** Hui Zhi, Hui Fu, Yunxin Zhang, Ni Fan, Chengcheng Zhao, Yunfei Li, Yujiao Sun, Yingpeng Li

**Affiliations:** ^1^ College of Chinese Materia Medica, Tianjin University of Traditional Chinese Medicine, Tianjin, China; ^2^ College of Integrated Chinese and Western Medicine, Tianjin University of Traditional Chinese Medicine, Tianjin, China; ^3^ Experimental Teaching and Practical Training Center, Heilongjiang University of Chinese Medicine, Harbin, China

**Keywords:** cGAS-STING pathway, traditional Chinese medicine, immunity, disease, cancer, infection6 cGAS-STING pathway, infection

## Abstract

The cGAS-STING signaling pathway is a critical component of the innate immune response, playing a significant role in various diseases. As a central element of this pathway, STING responds to both endogenous and exogenous DNA stimuli, triggering the production of interferons and pro-inflammatory cytokines to enhance immune defenses against tumors and pathogens. However, dysregulated activation of the STING pathway is implicated in the pathogenesis of multiple diseases, including autoinflammation, viral infections, and cancer. Traditional Chinese Medicines (TCMs), which have a long history of use, have been associated with positive effects in disease prevention and treatment. TCM formulations (e.g., Lingguizhugan Decoction, Yi-Shen-Xie-Zhuo formula) and active compounds (e.g., Glabridin, Ginsenoside Rd) can modulate the cGAS-STING signaling pathway, thereby influencing the progression of inflammatory, infectious, or oncological diseases. This review explores the mechanisms by which TCMs interact with the cGAS-STING pathway to regulate immunity, focusing on their roles in infectious diseases, malignancies, and autoimmune disorders.

## Graphical Abstract

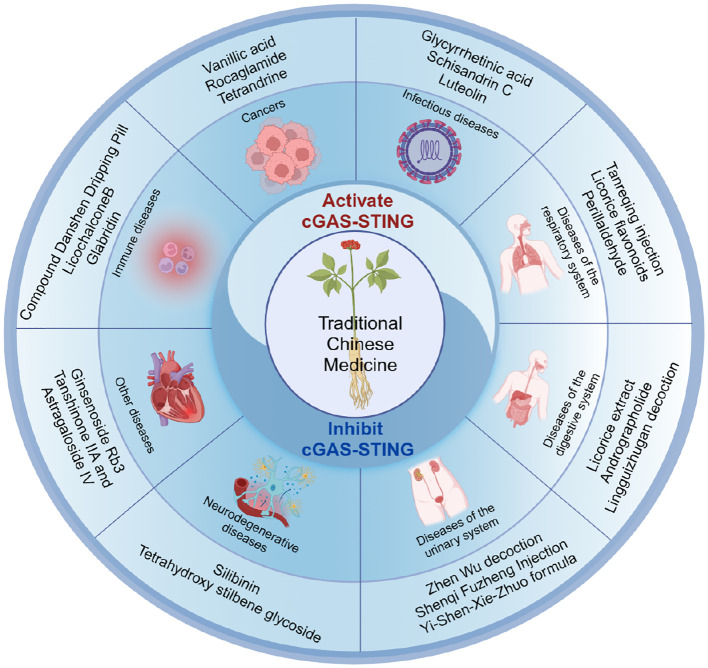

## Highlights

- Traditional Chinese Medicine (TCM) has a rich history of preventing and treating many diseases. This review explores how TCM modulates the cGAS-STING signaling pathway and its therapeutic potential.
- To elucidate the intricate relationship between the STING pathway and different diseases, and to analyze TCM as a potential agonist or inhibitor of the STING pathway.
- By conducting an extensive literature review, we explore the key proteins within the cGAS-STING pathway and their significance as biomarkers in TCM-based immunomodulation and the treatment of various diseases.

## 1 Introduction

Immune system homeostasis is vital to overall health, as proper immune regulation ensures normal physiological functions, while dysregulation can lead to various diseases (1, 2). The innate immune system play a crucial role in recognizing pathogen-associated molecular patterns and danger-associated molecular patterns through pathogen recognition receptors. These receptors form as the first line of defense against bacterial and viral infections, as well as aseptic inflammatory, by triggering the production of pro-inflammatory and anti-viral cytokines (3).

The cGAS-STING signaling pathway, as an important element of innate immunity, has garnered significant attention in recent years for its role in maintaining immune system homeostasis (4). This pathway plays a crucial role in antitumor immunity, and inflammatory and infectious diseases, as it recognizes various sources of cytoplasmic DNA, including bacterial, viral, and mitochondrial DNA (5). Upon detection of cytoplasmic DNA, cGAS generates cyclic GMP-AMP (2′3′-cGAMP), which activates STING. This process leads to TANK-binding kinase 1 (TBK1) phosphorylation (pTBK1) and induces type I interferon (IFN-I) transcription (6–8). In antiviral infections, STING acts through an IFN-I-driven immune response (7, 9). Activation of cGAS-STING enhances the ability of immune cells to target antigens through multiple pathways, thereby defending against pathogen invasion (10, 11). However, structural or functional abnormalities in this pathway may also contribute to the development of autoimmune and inflammatory diseases, such as systemic lupus erythematosus (SLE) and non-alcoholic fatty liver disease (NAFLD). Although cGAS is able to sense double-stranded DNA (dsDNA), it is unable to distinguish between its own DNA and exogenous DNA (12). Prolonged stimulation of aberrant DNA can lead to the overactivation activation of the STING pathway, resulting in excessive synthesis and release of IFN-I and inflammatory cytokines, which drive the progression of inflammatory or autoimmune diseases (13). In addition, tumor-derived dsDNA activates the STING pathway in antigen-presenting cells, promoting interferon production. This process facilitates dendritic cell maturation, T cell recruitment, and enhances anti-tumor responses (14).

Traditional Chinese Medicine (TCM) has a long history, traditionally employed for the prevention and treatment of a wide spectrum of diseases. Studies have shown that TCM has a bidirectional regulatory effect in immunomodulation, both activating the immune system and suppressing excessive immune responses (15). For example, ginseng contains a variety of active ingredients (e.g., ginsenosides, ginseng polysaccharides) that have immunomodulatory effects. A randomized controlled trial showed that ginseng polysaccharide for 8-14 weeks enhanced the cytotoxic activity of NK cells and up-regulated serum TNF-α levels (16). TCM has been associated with potential benefits in adjunct cancer therapy, particularly in alleviating clinical symptoms, extending patient survival, and modulating immune functions (17–19). TCM principles identify ‘yang deficiency’ as the underlying cause of breast cancer. The classic anti-neoplastic formula, Yanghe Decoction (YHD), has been shown in contemporary research to decrease myeloid-derived suppressor cells (MDSCs) and suppress the tumor microenvironment’s iNOS and ARG-1 expression. At the same time, it enhances the immune response by increasing the number of natural killer T cells (NKTs) and CD4 T cells (20). Unlike chemotherapy, traditional Chinese medicine (TCM) not only directly targets tumor cells but also effectively boosts the immune response. For instance, the TCM formula Shugan Jianpi Decoction has been shown to suppress the proliferation of MDSCs and enhance the inflammatory regulatory functions of NKT cells (21). In addition, certain herbal components, such as astragaloside derived from Huangqi (*Radix Astragali*), can significantly promote IFN-γ secretion from T cells and enhance T cell immunoreactivity (22).

**Figure 1:**
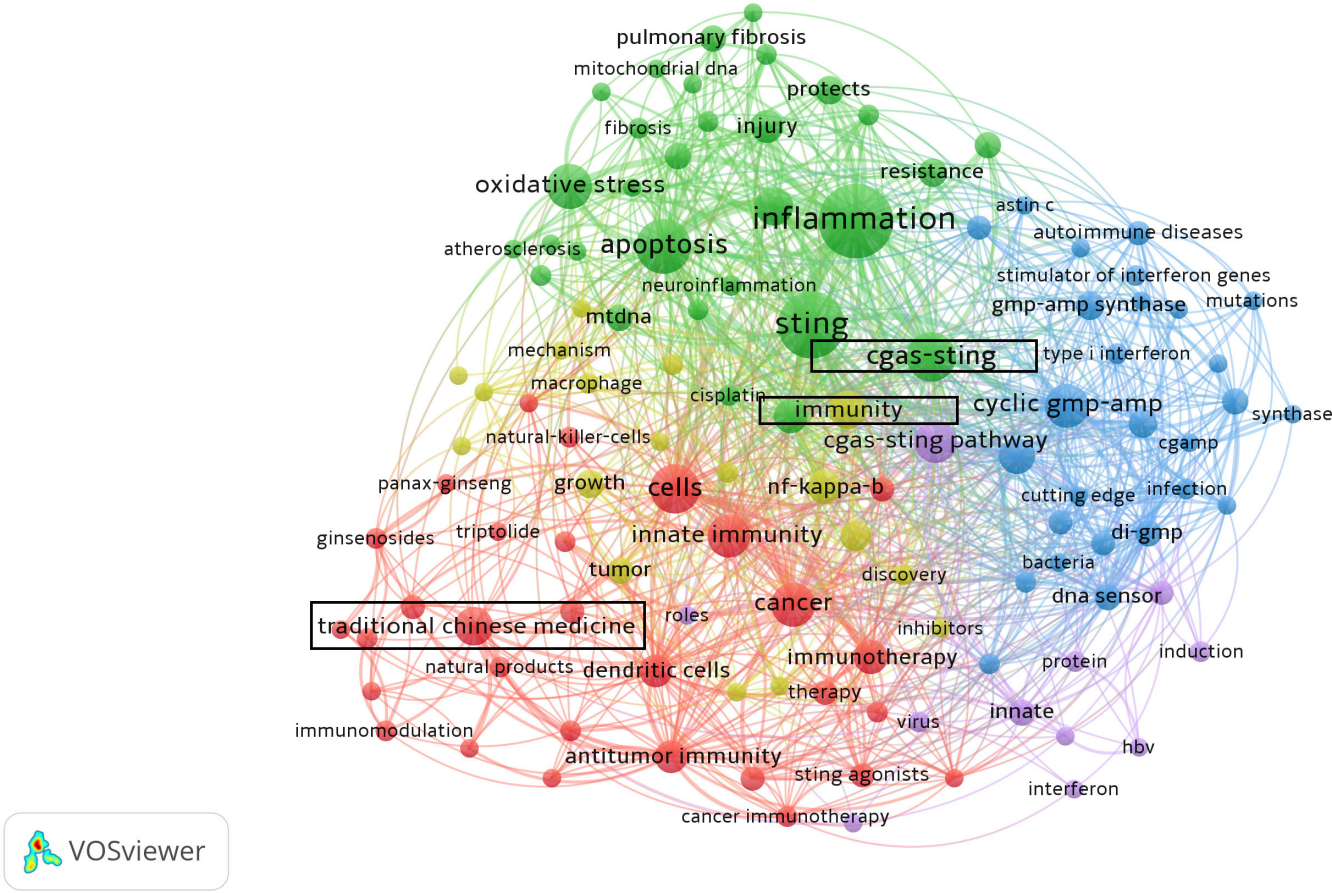
A VOSviewer analysis of key terms related to TCM and the cGAS-STING signaling pathway underscores their significance as prominent and actively explored research areas. Created with VOSviewer.

Increasing attention has been given to the immunomodulatory potential of TCM, particularly the ability of TCM active compounds or formulations to address diseases such as inflammation, infection, and cancer, by modulating the cGAS-STING signaling pathway (**Figure 1**). By regulating this pathway, Chinese medicines may enhance the body’s immune response to pathogens and tumors, as well as inhibit excessive immune responses. However, these effects are primarily observed in preclinical studies investigating underlying mechanisms of disease treatment. Further well-designed clinical trials are necessary to confirm these findings.

Despite the growing recognition of the cGAS-STING signaling pathway’s significance in immunomodulation, comprehensive reviews on how TCMs target this pathway remain scarce. This paper aims to address this gap by summarizing recent advancements in understanding the cGAS-STING pathway’s role in immune regulation and the potential mechanisms underlying herbal interventions. The findings are expected to serve as a valuable reference for future in-depth mechanistic research.

## 2 Structural characterization of STING and its application in clinical therapy

Innate immunity serves as the body’s first line of defense against invading pathogens (23). The stimulator of interferon genes (STING) is a critical protein that mediates various DNA receptors in this system. STING, also referred to as ERIS, MYPS, and MITA, is a conserved transmembrane protein encoded by the TMEM173 gene. Predominantly localized in the endoplasmic reticulum, it is also found on Golgi and mitochondrial membranes (24). STING consists of 379 amino acids and contains an N-terminal transmembrane region and a C-terminal cytoplasmic globular structural domain, which interacts with another STING molecule to form an intact dimer (25).

**Figure 2:**
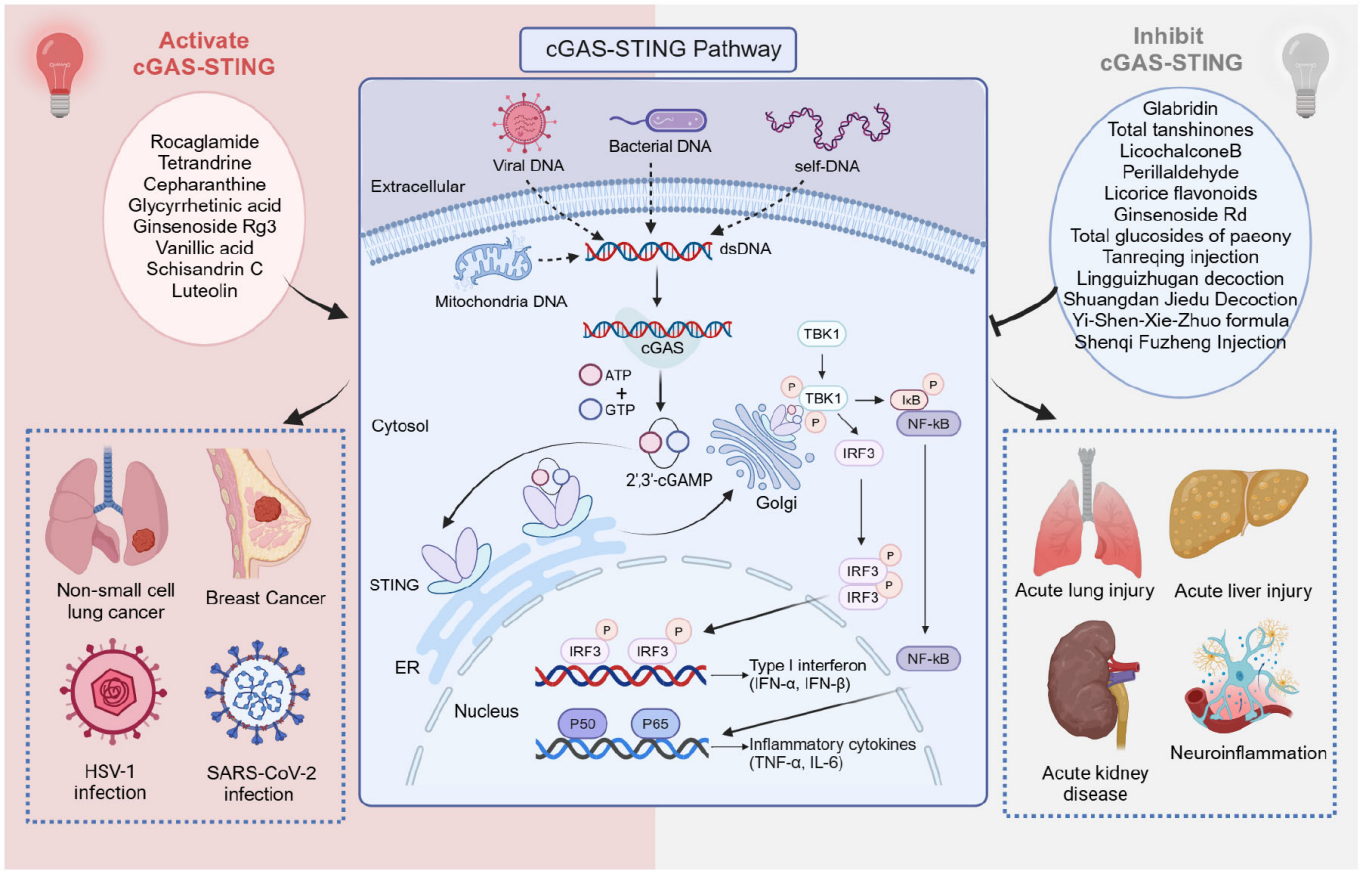
Mechanisms of cGAS-STING pathway activation, Chinese medicine inhibits or activates cGAS-STING signaling pathway to treat various diseases. Created with BioRender.com.

STING detects cytoplasmic dsDNA and serves as a direct sensor for endogenous cyclic dinucleotides (CDNs) (26). Activation of cGAS, a nucleotidyltransferase that senses cytoplasmic non-self DNA, catalyzes the production of 2′3′-cGAMP, a CDN composed of adenosine and guanosine (**Figure 2**) (27, 28). In addition to cGAMP, STING can be triggered by bacterial-derived CDNs, such as cyclic di-AMP and cyclic di-GMP (29). CDNs and 2′3′-cGAMP bind to STING in the endoplasmic reticulum, facilitating the dimerization and translocation of STING to the perinuclear region (29, 30). STING recruits TBK1 and IκB kinase (IKK) during translocation, which then relocates to the perinuclear region. These kinases phosphorylate interferon regulatory factor 3 (IRF3) and nuclear factor-κB (NF-κB), which activates the expression of IFN-I and pro-inflammatory cytokines (31–33). IFN-I enhances immune responses by promoting the activation and function of immune cells such as dendritic cells, T cells, and natural killer cells (34).

The cGAS-STING pathway is a conserved innate immune mechanism that responds to pathogenic infections, DNA damage, and aberrant cell activities like uncontrolled replication or senescence (35). In cancer therapy, activation of STING enhances tumor antigen presentation and promotes antitumor immunity, making it an attractive immunotherapeutic target (36). Studies have shown that cGAS-STING agonists not only induce tumor cell senescence but also boost adaptive anticancer immunity and combine efforts with immunotherapy (37, 38). Clinical trials have explored two main types of STING agonists: cyclic dinucleotides (CDNs, e.g., ADU-S100) and non-CDN (e.g., DMXAA) (39, 40). For instance, ADU-S100, the first CDN derivative in clinical trials, has demonstrated the ability to stimulate IFN-β production by human immune cells, showcasing its therapeutic potential (41). Additionally, cGAS activity is critical for the success of immune checkpoint blockade therapies, with STING agonists showing promise in enhancing vaccine efficacy for tumors resistant to PD-1 inhibitors (42).

In addition to cancer therapy, the cGAS/STING pathway plays a vital role in viral infections. Many DNA viruses, such as herpes simplex virus (HSV) and hepatitis B virus (HBV), are able to inhibit viral replication by initiating IFN-I production through activation of this pathway (43, 44). This suggests that STING agonists may have broad-spectrum antiviral potential (45). For example, DMXAA is a potent antiviral agent in mice; α-Mangostin, a flavonoid with antimicrobial properties, has been shown to possess antiviral properties and anti-DENV and HBV replicative activity in cellular experiments (46–48). However, numerous viruses such as HSV, Human CMV (HCMV), etc. have evolved mechanisms to circumvent this pathway (49–51). For instance, Epstein-Barr virus (EBV) suppresses localized innate immunity by targeting STING for degradation through the E3 ubiquitin ligase TRIM29 (52).

However, despite its protective roles, excessive activation of STING can lead to autoimmune conditions like Aicardi-Goutières syndrome (AGS) and SLE (53–55). In these diseases, both autologous DNA and mitochondrial DNA may be misrecognized by cGAS, leading to the activation of STING and an excessive IFN-I response (56). To address this, inhibitors targeting STING or cGAS have emerged as therapeutic candidates. Compounds like Acrinamin and Oxychloroquine show potential in blocking cGAS activation, offering new avenues for treating autoimmune disorders (57).

Although several STING agonists and inhibitors have been developed, they have had limited effect in clinical translation. TCM offers a valuable resource for the development of STING modulators, including inhibitors and activators. This underscores TCM’s potential to address various diseases through immunomodulatory mechanisms, providing a robust foundation for future research and drug development (**Figure 2**).

## 3 Role of Chinese medicines in immunomodulation

Chinese medicines play a vital bidirectional role in immunomodulation, both activating the immune system and suppressing excessive immune responses. This regulation is achieved by influencing various aspects such as immune cells, cytokines, and immune organs. Studies have shown that certain Chinese medicines can effectively regulate the production of immune cells and cytokines (58, 59). Certain herbal medicines boost innate immune system activity, while others act on cellular subpopulations of adaptive immunity (60).

Chinese medicines act by enhancing the function of various immune cells, including macrophages, dendritic cells, NK cells, T cells, and B cells. For example, Ganoderma lucidum polysaccharide (PS-G), the primary active compound in Ganoderma lucidum, has been shown to promote activation and maturation of dendritic cells derived from human monocytes (61). Herbal medicines also regulate T lymphocyte activity by stimulating their proliferation and differentiation, promoting cytotoxic T lymphocyte production, and modulating the TH1/TH2 balance as well as the function of T helper (TH) cell subsets (59). For example, polysaccharides from *Cordyceps sinensis* enhance the expression of transcription factors such as T-bet, GATA-3, and RoR-γt in TH cells, thereby increasing the number of TH1, TH2, and TH17 cells (62). B lymphocytes, the main cells of humoral immunity, depend on antigen stimulation to release antibodies (63). Research indicates that polysaccharides from *Dendrobium huoshanense* and *Atractylodes macrocephala* Koidz significantly increase B lymphocyte populations, thereby strengthening humoral immunity (64, 65).

In addition, herbal medicines can regulate the production of specific cytokines, including interferons (IFN-α, IFN-β, IFN-γ), tumor necrosis factor (TNF-α), and interleukins (e.g., IL-1, IL-2, IL-4), which are pivotal in immune and inflammatory processes. For example, polysaccharides from *Atractylodes macrocephala* and *Astragalus membranaceus* markedly upregulated IFN-γ expression *in vitro* experiments (66, 67).

The diversity of immunomodulatory components in TCM provides a wide range of therapeutic potential for clinical applications. These components are mainly divided into two categories: anti-inflammatory and immune-enhancing. The anti-inflammatory category includes phenolic acids (e.g., vanillic acid, salvianolic acid B), flavonoids (e.g., luteolin, glabridin), volatile oils (e.g., perillaldehyde, zingiber officinale), lignans (e.g., schisandrin C, asarinin), and alkaloids (e.g., rocaglamide, tetrandrine), while the immune-enhancing category mainly consists of polysaccharides (e.g., lycium barbarum polysaccharides, astragalus membranaceus polysaccharides) and glycosides (e.g., ginsenoside Rg3, ginsenoside Rd) (68–72). These components regulate the body’s immune response through different pathways, enabling TCM to demonstrate unique advantages in the treatment of immune-related diseases.

Additionally, TCM is closely related to the concept of “medicine and food,” i.e., certain species offer both nutritional benefits and therapeutic effects (73). With the growing emphasis on preventive care and holistic health in recent years, many TCM ingredients have been incorporated into daily diets as functional foods or dietary supplements and have become an important part of alternative therapies (74, 75).

## 4 The role of TCM in modulating the cGAS/STING pathway in clinical diseases

In recent years, activators and inhibitors of the cGAS-STING pathway have attracted widespread attention, but clinical translation still faces challenges. TCM, as a valuable cultural heritage of the Chinese nation, has shown promising potential in modulating immune-related diseases. Several active compounds have been found to effectively modulate the cGAS/STING signaling pathway and improve diseases. These include ginsenoside Rg3 and ginsenoside Rd, which are derived from *Panax ginseng*; glabridin and licochalcone B, obtained from *Glycyrrhiza uralensis*; perillaldehyde, isolated from *Perilla frutescens*; and schisandrin C, extracted from *Schisandra chinensis* (**Figure 2**). Natural products have been valued as indispensable resources for discovering novel therapeutic molecules and are instrumental in managing diseases (76–78). The mechanisms and clinical applications of TCM in modulating this pathway will be summarized below, categorized by different types of diseases.

### 4.1 Immune diseases

Normal activation of the cGAS-STING pathway can trigger immune responses and enhance the ability of immune cells to eliminate antigens and defend against pathogens. Nevertheless, excessive or abnormal activation of this pathway may trigger immune dysregulation, which in turn leads to the development of autoimmune diseases such as SLE and NAFLD. Research has demonstrated that TREX1 deficiency has a close association with various autoimmune diseases (e.g., AGS, SLE) and that in TREX1-deficient mouse models, deletion of cGAS or STING can ameliorate these disease phenotypes (79–81).

TCM has demonstrated promise in modulating the cGAS-STING pathway. For instance, total tanshinones, the main active ingredient of Salvia miltiorrhiza, can block STING-IRF3 binding, thereby suppressing aberrant pathway activation and alleviating autoimmune conditions associated with TREX1 deficiency TREX1 deficiency (82). Perillaldehyde (PAH), another TCM ingredient, is a natural monoterpenoid extracted from Perilla frutescens, has demonstrated the ability to inhibit STING pathway activation significantly (83). By targeting cGAS proteins, PAH reduces the interferon response, offering a potential therapeutic approach for cGAS-mediated autoimmune diseases (84).

Glabridin, an active ingredient in licorice, specifically inhibits the cGAS-STING pathway by decreasing the levels of IFN-I, IL-6, and TNF-α, thereby alleviating immune disorders triggered by TREX1 deficiency (85). In addition, Licochalcone B and Licochalcone D also showed significant anti-inflammatory effects by inhibiting STING downstream signaling and improved symptoms of inflammatory diseases, such as colitis, in experimental models (86, 87).

In addition, Compound Danshen Dropping Pills, widely utilized for managing cardiovascular conditions such as angina pectoris and acute myocardial infarction, have successfully completed Phase III clinical trials with the U.S. Food and Drug Administration (88–90). This TCM formulation has shown efficacy in reducing multi-organ inflammatory responses in TREX1-deficient mice by disrupting STING-TBK1 interactions and blocking cGAS-STING pathway activation, highlighting its therapeutic potential for inflammatory conditions, including obesity-induced insulin resistance (91).

### 4.2 Cancers

Tumorigenesis is a complex, multistep process, and conventional cancer research usually focuses on a single target (92). However, due to their diversity and complexity, the therapeutic effects are often limited. TCM has unique advantages in tumor therapy through holistic regulation and multi-target intervention (93, 94).

Ginsenoside Re, derived from ginseng, can regulate the host immune system and exert anticancer effects through multiple pathways (95). In non-small cell lung cancer (NSCLC), ginsenoside Re exerts antitumor effects by inhibiting the epithelial-mesenchymal transition (EMT) process. It does so through the inhibition of the AMPKα1/STING positive feedback loop and the reduction of M2-like macrophage formation (96). In addition, Rocaglamide (RocA), a compound extracted from Aglaia odorata, promotes the leakage of mitochondrial DNA (mtDNA) into the cytoplasm and activates the cGAS-STING pathway. This process increases tumor infiltration of NK cells and significantly enhances anti-tumor immunity in NSCLC (97).

Tetrandrine, derived from *Stephania tetrandra* S. Moore, is a bisbenzylisoquinoline alkaloid with the ability to inhibit tumor proliferation and angiogenesis (98). Tetrandrine activates the STING/TBK1/IRF3 pathway, promoting CCL5 and CXCL10 production. This enhances the infiltration of macrophages, dendritic cells, and CD8 T cells in the tumor microenvironment, significantly inhibiting the growth of NSCLC (99).

Vanillic acid is a phenolic compound present in TCMs such as Angelica sinensis and ginseng, with antioxidant and antimicrobial properties (100). It promotes macrophage polarization to the M1 type through activation of the STING pathway and enhances tumor cell apoptosis and anti-tumor immune response (101).

Breast cancer is a common tumor in women with high morbidity and mortality rates (102). Formononetin, an active ingredient in red clover and astragalus, inhibits the proliferation of BC cells by interfering with PD-L1 and inhibiting the activation of the STING-NF-κB pathway (103). Ginsenoside Rg3 inhibits tumor growth by inhibiting angiogenesis, inducing apoptosis, and other mechanisms. When combined with STING agonists, Rg3 can induce tumor-associated macrophages to polarize from M2 to M1 and improve the tumor microenvironment, effectively inhibiting the growth and invasion of triple-negative breast cancer (104).

### 4.3 Infectious diseases

The cGAS-STING pathway has played a crucial antiviral role during evolution, and its activation is closely linked to antiviral cellular responses (105). cGAMP synthesis is the critical first step in initiating cGAS-mediated antiviral effects. The downstream effects mainly include the synthesis of antiviral type I interferon and related genes (106, 107). TCM provides a rich source of natural compounds, and many herbs show antiviral, anti-inflammatory, and immunomodulatory effects, making them potential candidates for the development of antiviral drugs.

*Schisandra chinensis* (Turcz.) Baill., a long-established TCM, has been shown to modulate host immunity and exhibit anticancer, antiviral, and hepatoprotective effects (108, 109). Its active ingredient, Schisandrin C, was found to inhibit HBV replication by promoting the interaction between TBK1 and STING, enhancing the activation of the cGAS-STING pathway and promoting the expression of IFN-β and interferon-stimulated genes (110). Another active ingredient is luteolin, a natural flavonoid found in various plants (111). Research indicates that luteolin combats HSV-1 by activating the cGAS-STING pathway, thereby enhancing antiviral interferon production (112). Liuwei Wuling Tablet consists of various ingredients, including Schisandra chinensis and chasteberry, which have been shown to nourish the kidneys and liver while also exhibiting antiviral activity (113). The combination of Schisandrin C and Luteolin has been found to inhibit HBV replication and attenuate HBV infection by activating the cGAS-STING pathway (114).

Glycyrrhetinic acid (GA), a major constituent of licorice, exhibited anti-inflammatory, antioxidant, and antiviral effects during the COVID-19 pandemic (115, 116). GA was found to inhibit SARS-CoV-2 infection by activating cGAS-STING pathway (117). Cepharanthine (CEP) has demonstrated inhibitory effects against viruses such as HIV, SARS, and HSV-1 (118). CEP promotes cellular autophagy, thereby inhibiting HSV-1 infection (119). Euphorbia fischeriana Steud is a perennial herb whose root has traditionally been utilized in TCM to treat diseases such as cancer, edema, and ascites. Dpo, a compound isolated from the root of E. fischeriana, has been found to activate antiviral innate immune responses by targeting STING and utilizing the IRFs/ELF4 pathway (120). Similarly, Ginsenoside Rg3 has been shown to stimulate a type I interferon response via the cGAS-STING signaling axis. This response is supported by gut-derived short-chain fatty acids like acetate and propionate, offering protection against enteroviral infections (104).

In the context of sepsis—a severe systemic inflammatory condition triggered by bacterial or fungal infections and often leading to multiple organ dysfunction—Glycyrrhiza uralensis polysaccharides have demonstrated protective effects. These are achieved by disrupting the interactions between STING, TBK1, and IRF3, thereby reducing cGAS-STING pathway activation and mitigating sepsis-related damage (121).

### 4.4 Diseases of the respiratory system

Acute lung injury (ALI) is a serious lung disease recognized globally, manifesting as a persistent acute inflammatory response that is associated with high morbidity and mortality (122). Despite significant advances in therapy, treating ALI remains a major clinical challenge. The cGAS-STING pathway plays a vital role in the pathogenesis of ALI, affecting immune response, apoptosis, vascular permeability, and oxidative stress, which exacerbate inflammation and tissue damage (123). Various herbal medicines can improve ALI or pulmonary fibrosis by modulating this pathway (**Table 1**).

**Table 1:** Traditional Chinese medicine can treat or alleviate respiratory diseases by regulating the cGAS-STING signaling pathway.

Compounds/single TCM	Origins	Molecular formula	Cells/Animals	Mode of administration	Doses	Course of treatment	Functions	Mechanisms	References
Licorice flavonoids	*Glycyrrhiza glabra L.*	–	BMDMs,THP-1 cells,HEK-293 Cells, C57BL/6J mice	Gavage	20 mg/kg, 40 mg/kg	10hours	Inhibition of the expression of type I interferons and related downstream genes, as well as inflammatory cytokines and TNF-α	Blocking of cGAMP synthesis	(124)
Perillaldehyde	*Perilla frutescens* (L.) Britt.	C_10_H_14_O	RAW264.7 cells, C57BL/6 mice	Intraperitoneal injection	50 mg/kg, 10 0 mg/kg, 2 0 0 mg/kg	24hours	Inhibiting LPS-induced lung histological changes, inflammatory cell infiltration and oxidative stress	Inhibiting cGAS/STING-mediated IRF3/NF-κB signaling	(125)
Apigenin	*Matricaria chamomilla* L.,Perilla frutescens (L.) Britt.	C_15_H_10_O_5_	THP-1 cells,HEK293T cells, C57BL/6 mice	Intraperitoneal injection	50 mg/kg	24hours	Alleviating innate immune responses and mitigating inflammation in LPS-induced ALI	Inhibits STING expression, reduces dimerization, phosphorylates nuclear translocation of IRF3, and disrupts the association between STING and IRF3, IFNβ1↓	(126)
Ursodeoxycholic acid	*Selenaretos thibetanus* Cuvier	C_24_H_40_O_4_	C57BL/6 mice	Oral pre-treatment	30 mg/kg, 60 mg/kg	24hours	Inhibiting pulmonary edema, inflammatory cell infiltration, pro-inflammatory cytokines production, and oxidative stress	Blocking PANoptosis-like cell death via STING pathway	(127)
Shuangdan Jiedu Decoction	*Lonicera japonica* Thunb,*Forsythia suspensa* (Thunb.) Vahl,*Paeonia lactiflora* Pall,*Salvia miltiorrhiza* Bunge,*Paeonia suffruticosa* Andr,*Glycyrrhiza uralensis* Fisch	–	BMDMs,THP-1 cells, C57BL/6 mice	Gavage	3.7 g/kg,7.4 g/kg,0.98 g/kg	12hours	Alleviating LPS-induced ALI by suppressing the levels of proinflammatory cytokines, and the number of neutrophils, decreasing the inflammatory factor-associated gene expression	Inhibit multiple stimulus-driven activation of cGAS-STING and inflammasome	(128)
Tanreqing injection	*Scutellaria baicalensis* Georgi,*Selenaretos thibetanus* Cuvier,*Capra hircus* Linnaeus,*Lonicera japonica* Thunb.,*Forsythia suspensa* (Thunb.) Vahl	–	RAW 264.7 cell,Mouse bone marrow neutrophils, C57BL/6 mice	Intraperitoneal injection	2.6 ml/kg,5.2 ml/kg	6hours	Inhibiting inflammatory responses and oxidative stress	Downregulating STING signaling pathway	(129)
			C57BL/6 mice	Intraperitoneal injection	2.6 ml/kg,5.2 ml/kg	21 days	Inhibiting inflammatory responses and reducing the occurrence of fibrosis	Inhibiting STING-mediated endoplasmic reticulum stress signaling pathway	(130)
20(S)-Protopanaxadiol	*Panax ginseng* C. A. Mey.	C_30_H_52_O_3_	MLE-12 cell, C57BL/6 mice	Gavage	10 or 40 mg/kg	2 weeks	Inhibiting inflammatory responses and reducing the occurrence of fibrosis	Inhibiting STING expression by activating AMPK	(131)

For example, licorice flavonoids possess anti-inflammatory activity and inhibit cGAMP synthesis, thereby preventing overactivation of the cGAS-STING pathway and ameliorating lipopolysaccharide (LPS)-induced ALI (124). Perillaldehyde alleviated acute lung injury by inhibiting the cGAS-STING-mediated IRF3/NF-κB pathway (125). Additionally, apigenin and ursodeoxycholic acid (UDCA) have demonstrated efficacy in alleviating ALI by inhibiting STING-related signaling pathways. Apigenin attenuates the LPS-induced inflammatory response by inhibiting the STING/IRF3 pathway, whereas UDCA mitigates sepsis-induced lung injury by blocking cell death via the STING pathway (126, 127). Traditional Chinese medicine compound preparations, such as Shuangdan Jiedu Decoction and Tanreqing injection (TRQ), have also significantly ameliorated LPS-induced ALI and other respiratory-related diseases by regulating the STING pathway through multiple mechanisms (128, 129). TRQ is a proprietary Chinese medicine that is commonly used for lung diseases such as pneumonia and idiopathic pulmonary fibrosis (IPF) (132–134). Clinical evidence suggests that TRQ can alleviate the development of pulmonary fibrosis and improve lung function in patients (130). Recent studies have shown that 20(S)-Protopanaxadiol, isolated from ginseng, and TRQ can improve pulmonary fibrosis by modulating the cGAS-STING pathway (130, 131).

### 4.5 Diseases of the digestive system

Liver fibrosis is a chronic liver disease triggered by various factors, including excessive alcohol consumption, viral infections (HBV and HCV), and non-alcoholic steatohepatitis (NASH) (135–137). Recent studies have shown that the cGAS-STING pathway plays an important role in the pathological process of liver fibrosis, and various traditional Chinese medicines can exert anti-fibrotic effects by regulating this pathway.

Naringenin, an anti-inflammatory flavonoid extracted from citrus plants, has been shown to directly bind to cGAS (138). It reduces inflammatory factors secreted by hepatic stellate cells by inhibiting the cGAS-STING pathway, thereby alleviating liver fibrosis (139). Licorice extract improved hepatic inflammation and fibrosis in a mouse model of NASH, with its mechanism of action including inhibition of the cGAS-STING pathway (140). Oroxylin A, a baicalin derivative, activated the cGAS-STING pathway, promoted the secretion of cytokine IFN-β, induced hepatic stellate cell senescence, and acted as an antifibrotic agent (141).

Modulation of the cGAS-STING pathway by TCM can also alleviate acute liver injury. For example, total glucosides of paeon, on the other hand, reduced hepatic inflammation in an acute liver injury (ALI) model by inhibiting the STING-IRF3 interaction (142). Ginsenoside Rd protects mice from CCl_4_-induced ALI by inhibiting the cGAS-STING pathway and reducing iron death (143).

Lingguizhugan Decoction (LGZG) is a traditional Chinese herbal decoction that has been used for many years in the treatment of metabolic disorders and has been effective in alleviating obesity and dyslipidemia (144, 145). LGZG significantly reduced high-fat diet (HFD)-induced hepatic lipid deposition by inhibiting the STING-TBK1-NF-κB pathway in hepatic macrophages (146).

Additionally, drug-induced liver injury is a leading cause of acute liver injury and liver transplantation (147). Studies have shown that jujuboside B ameliorated acetaminophen-induced liver injury by upregulating Nrf2 protein expression and inhibiting the cGAS-STING pathway (148). Similarly, rhodopsin protected hepatocytes from APAP-induced toxicity by regulating Nrf2 and NLRP3 inflammatory vesicles, while inhibiting the cGAS-STING pathway (149).

Andrographolide, derived from Andrographis paniculata, has been shown to ameliorate chemotherapeutic drug-induced gastrointestinal mucosal inflammation by down-regulating the cGAS-STING pathway (150). Naringin can also attenuate intestinal ischemia-reperfusion injury by inhibiting the cGAS-STING pathway (151).

### 4.6 Diseases of the urinary system

Acute kidney injury (AKI) is a global health problem. Although cisplatin is an effective chemotherapeutic agent, its nephrotoxicity limits clinical use (152). Therefore, there is a need for nephroprotective drugs that are safe and do not compromise the antitumor effect. TCMs are widely used for preventing and treating renal diseases. From the Western medicine perspective, cisplatin triggers AKI primarily due to drug toxicity or edema, while from the TCM perspective, its pathogenesis involves spleen and kidney qi deficiency, damp-heat underflow, and blood stasis (153). Various traditional Chinese medicines and compound formulas can effectively alleviate cisplatin-induced AKI by regulating the cGAS/STING pathway.

Yi-Shen-Xie-Zhuo formula (YSXZF) is a Chinese herbal formula composed of four herbs: *Astragali Radix* (Huangqi), *Alismatis Rhizoma* (Zexie), *Paeoniae Radix Rubra* (Chishao), *Sargassum* (Haizao). Studies have shown that YSXZF can inhibit the cGAS/STING pathway, reduce the expression of inflammatory factors such as TNF-α, IL-3, and IL-1β, and decrease IRF1 activity, which in turn reduces the inflammatory response and prevents acute kidney injury (154). Shenqi Fuzheng Injection (SQFZ) consists of extracts from Codonopsis Radix and Astragali Radix, both of which possess anti-tumor and anti-inflammatory effects. It has been found that SQFZ can effectively inhibit the cGAS/STING pathway, attenuate cisplatin-induced nephrotoxicity, and improve the effectiveness of chemotherapeutic agents (155).

To improve the bioavailability of active ingredients in traditional Chinese medicine, recent studies have explored the use of nanotechnology. For example, baicalein (5,6,7-trihydroxyflavone, BA) possesses antioxidant and antitumor effects, but its poor water solubility and low bioavailability limit its clinical application. Self-assembly of silk fibroin peptide (SFP) into nanofibers encapsulating baicalein (SFP/BA NFs) enhances its *in vivo* efficacy, inhibits cisplatin-induced DNA damage and cGAS/STING pathway activation, and exerts a nephroprotective effect to prevent AKI (156).

Similarly, naringenin (NGN) has poor water solubility, limiting its application. To address this, researchers have developed NGN-loaded silk fibroin peptide nanofibers (SFP/NGN NFs). Cisplatin-induced mitochondrial damage leads to the release of mtDNA and activation of the cGAS-STING pathway, which in turn triggers the expression of inflammatory factors, such as IL-6 and TNF-α. SFP/NGN NFs effectively attenuated cisplatin-induced acute kidney injury by facilitating mitochondrial autophagy, decreasing the release of mtDNA and inhibiting the cGAS-STING pathway (157).

In addition, Zhen Wu decoction (a prescription composed of five herbs: Radix *Aconiti lateralis Preparata*, Poria, Radix *Paoniae alba*, ginger, and Rhizoma *Atractylodis macrocephalae*, which are decocted together for extraction) inhibited renal fibrosis by activating NRF2 and TFAM in renal tubules and promoting mitochondrial bioenergy production (158).

### 4.7 Neurodegenerative diseases

Neurodegenerative diseases are a group of chronic neurological disorders characterized by a progressive loss of neurons and an abnormal accumulation of specific proteins in the brain, accompanied by a decline in cognitive and motor function (159). This group includes Alzheimer’s disease (AD), Parkinson’s disease (PD), multiple sclerosis (MS), and amyotrophic lateral sclerosis (ALS) (160). Among these, Alzheimer’s disease (AD) is the most common neurodegenerative disorder worldwide, manifesting as severe cognitive decline (161).

As an anti-aging traditional Chinese medicine, Polygonum multiflorum has received widespread attention for its role in diseases such as AD, PD, and MS (162). Studies have shown that tetrahydroxy stilbene glucoside (TSG), the main active ingredient of Polygonum multiflorum, possesses significant anti-inflammatory, anti-aging, and memory-improving effects (163). TSG prevents neuroinflammation by modulating the cGAS-STING pathway, leading to significant improvement in cognitive decline in AD patients. In addition, TSG can reduce the formation of NLRP3 inflammatory vesicles by inhibiting the activation of the cGAS-STING pathway, thereby reducing the neuroinflammatory response and demonstrating its potential therapeutic value in Alzheimer’s disease (164).

Silibinin, an active ingredient extracted from the TCM silymarin, has attracted attention for its neuroprotective effects in AD models. Research has found that silibinin administration, downregulated the levels of IL-1β, TNF-α and IFN-β, as well as STING and IRF3, ameliorating depression/anxiety-like behaviors of Parkinson’s disease mouse model (165). While these findings suggest that silibinin may modulate the cGAS-STING pathway, it is important to note that the inhibition of pro-inflammatory cytokines such as IL-1β and TNF-α could also involve other signaling pathways, including NLRP3 inflammasome activation, NF-κB signaling, and the MAPK pathway (166, 167). And silibinin exerts significant neuroprotective effects by downregulating iron death injury and STING-mediated neuroinflammation, particularly in the STZ-induced sporadic AD model. This provides an important basis for silymarin as a potential drug for the treatment of AD (168). Given the multi-target nature of TCM, further studies are needed to clarify the mechanisms underlying silibinin’s effects on neuroinflammation and behavioral outcomes.

### 4.8 Other diseases

In addition to autoimmune diseases, tumors, and viral infections, a variety of Chinese herbal medicines can ameliorate other diseases by impacting the cGAS-STING pathway, potentially in conjunction with other molecular targets. For example, atherosclerosis is a chronic inflammatory disease of the arterial lining (169). Tetrandrine was found to inhibit the STING/TBK1/NF-κB pathway, reducing inflammation in macrophages attacked by oxidized low-density lipoprotein, and attenuating atherosclerosis in HFD-fed ApoE mice (170).

Myocardial ischemia-reperfusion injury (MIRI) is a major challenge in the treatment of acute myocardial infarction, primarily caused by oxidative stress and inflammatory responses induced by blood reperfusion (171, 172). *Astragalus membranaceus* (Fisch.) Bunge and *Salvia miltiorrhiza* Bunge are representative herbs used for replenishing Qi and activating blood circulation in traditional Chinese medicine, respectively. According to the compatibility theory of traditional Chinese medicine (173, 174), they are often used in combination (175). Astragaloside IV (As-IV) and Tanshinone IIA (Ta-IIA) are the primary active components of Astragalus membranaceus and Salvia miltiorrhiza, respectively. Research has indicated that the combined use of As-IV and Ta-IIA significantly reduces oxidative stress and apoptosis in cardiomyocytes by enhancing the inhibition of cGAS/STING signaling, thereby improving the therapeutic effect on MIRI (176).

In skin flap transplantation, ischemia/reperfusion (I/R) injury is the main cause of flap necrosis (177, 178). Ginsenoside Rb3, an active component of ginseng, has been shown to reduce leukocyte-endothelial cell adhesion and improve local microcirculation by inhibiting the phosphorylation of IRF3 in the STING pathway, effectively alleviating I/R injury in transregional flaps (179).

In addition, overactivation of the cGAS-STING pathway is closely related to cellular senescence. Liuwei Dihuang (LWDH), a classic Chinese herbal formula, shows potential for anti-endothelial cellular senescence. Studies have shown that LWDH reverses LPS-induced endothelial cell senescence by inhibiting the activation of the cGAS-STING pathway and blocking the interaction between JPX and STING. This provides a new approach for preventing and treating vascular endothelial cell aging (180).

In conclusion, TCM has demonstrated significant therapeutic potential in diseases such as atherosclerosis, myocardial ischemia-reperfusion injury, skin flap transplantation injury, and cellular senescence by modulating the cGAS/STING pathway. These studies provide a new scientific basis for the application of TCM in the treatment of modern diseases, as well as insights for the clinical development of more targeted TCM.

## 5 cGAS-STING pathway key proteins as biomarkers for TCM in immunomodulation and treatment of various diseases

Due to their multi-component and multi-target characteristics, traditional quality control methods have difficulty comprehensively assessing the safety and efficacy of Chinese medicines (181–183). To cope with these challenges, biomarkers have shown significant potential as tools for quality evaluation of TCM in recent years. Using technologies such as metabolomics, biomarkers can more comprehensively assess the systemic effects and compatibility of TCM. The components of schisandrol A, schisandrin A, gomisin N, and schisandrin B can be used as biomarkers for evaluating the quality standard of *Schisandra chinensis* (Turcz.) Baill (184). In addition, biomarkers can evaluate the clinical efficacy of TCM, such as NF2 and PPP1CA in CDDP, which are thought to be associated with its vasodilatory effects (185).

Biomarkers are equally important in disease treatment. In viral infections, IFN, a central factor in the antiviral response, has emerged as a potential therapeutic target for infections such as HCV and HBV (186). IL-6 plays an important role in the acute inflammatory response, and changes in its level correlate with the severity of infection. Especially in COVID-19, elevated IL-6 levels are closely associated with disease progression, suggesting its potential as a marker for monitoring treatment efficacy (187).

The cGAS-STING pathway is an important part of the innate immune system and has emerged as a potential therapeutic target for a variety of diseases in recent years (188–190). cGAS recognizes intracellular DNA and activates STING proteins, which in turn initiates downstream signaling pathways and induces antiviral and pro-inflammatory factors (191). cGAS-activated signaling molecules, such as TBK1 and IRF3, play a key role in immunoregulation (10). These proteins are not only key regulators of disease progression, but they may also be important biomarkers for evaluating therapeutic effects.

Traditional Chinese medicine plays an immunomodulatory role in the treatment of many diseases by modulating the cGAS-STING pathway. For example, total glucosides of paeony can alleviate liver inflammation caused by acute liver injury by inhibiting the STING-IRF3 interaction (142). Tetrahydroxy stilbene glucoside from Polygonum multiflorum was found to reduce neuroinflammation by inhibiting the cGAS-STING pathway, thereby improving cognitive function in patients with Alzheimer’s disease (164). In addition, ginsenoside Rb3 was effective in ameliorating ischemia-reperfusion injury in skin flap transplantation by inhibiting the STING-mediated inflammatory response (179). These studies demonstrated the modulatory effects of TCM on key proteins in the cGAS-STING pathway, suggesting that these proteins can be used as biomarkers of TCM therapy for assessing efficacy and potential for individualized treatment.

In summary, key proteins such as cGAS, STING, TBK1, and IRF3 play important roles in the occurrence and development of diseases. By regulating the expression and activity of these proteins, TCM can effectively regulate immune responses and treat a variety of diseases. Therefore, the key proteins in the cGAS-STING pathway can not only be used as targets for TCM to regulate immune and inflammatory responses, but they also have the potential to serve as biomarkers for clinical therapeutic effects. This provides a new direction for the future application of TCM in precision medicine.

## 6 Summary and prospect

TCM holds an important position in the field of medicine due to its unique bidirectional immunomodulatory ability, which can activate the immune system to enhance the body’s defense, while also moderately inhibiting excessive immune responses and reducing inflammation and autoimmune diseases. The cGAS-STING pathway, an important component of the innate immune system, plays a key role in defending against viral and bacterial infections, modulating cellular damage, inflammatory responses, autophagy, and tumor immunity (56, 192). Therefore, the cGAS-STING pathway has become a potential drug target for treating inflammatory diseases, tumors, and immune dysregulation. TCM has shown unique potential in modulating this pathway, providing new strategies for the treatment of a variety of diseases.

Currently, the development of activators and inhibitors of the cGAS-STING pathway is a research priority. Although STING agonists have shown promising results in preclinical antitumor studies, their clinical translation faces many challenges. For example, modified CDN compounds are rapidly degraded *in vivo* due to poor metabolic stability, which affects the durability of their efficacy (193). In addition, the low cellular uptake rate of CDN makes it difficult for the drug to efficiently enter target cells, which in turn limits its antitumor effects (194). While most studies rely on intra-tumor drug delivery, there is a lack of delivery technologies that can be applied on a large scale, further limiting the potential application of STING agonists in clinical therapy. In addition, the limited targeting of STING agonists may lead to off-target effects, triggering unnecessary immune activation and increasing autoimmune risks (195). Thus, improving the targeting and safety of STING agonists remains a critical issue for realizing their clinical applications. Regarding STING inhibitors, although compounds such as H-151, C-176, BB-Cl-amidine, and sulforaphane have been reported to inhibit the activation of the cGAS-STING pathway, their therapeutic potential remains limited (196–200). H-151, as the most promising STING inhibitor, inhibits palmitoylation by binding to the Cys91 site of the STING protein (196). However, studies on it are still at the animal experiment stage. Therefore, the development of clinically applicable STING inhibitors in inflammatory and autoimmune diseases remains an urgent topic.

Chinese medicines show remarkable potential in modulating the cGAS-STING pathway, especially in the treatment of inflammatory diseases. For example, compounds such as perilla aldehyde, ursodeoxycholic acid, total glucosides of paeony, and andrographolide affect the activity of this pathway through different mechanisms. Perillaldehyde has been shown to inhibit the innate immune response induced by cytosolic DNA by inhibiting cGAS activity and to attenuate the inflammatory response by reducing the release of inflammatory factors through inhibition of downstream signaling after STING activation. Ursodeoxycholic acid, on the other hand, inhibits the production of pro-inflammatory cytokines by blocking PANoptosis-like cell death through inhibition of the STING pathway. Various active components in licorice, such as glabridin, licorice flavonoids, and licorice chalcone B, can inhibit cGAS-STING-mediated inflammatory responses by modulating the cGAS-STING pathway, thereby exerting therapeutic effects on inflammatory diseases. TRIM29 has been reported to contribute to the pathogenesis of viral myocarditis by enhancing ROS-mediated oxidation of TBK1, thereby inhibiting its function (201). Both TRIM29 and TRIM18 play pivotal roles in the progression of various virus infections, including viral enteritis, viral myocarditis, and various organ inflammations (52, 202, 203). Studies suggest that TCM, with its rich repertoire of antiviral herbal compounds (such as quercetin and ginsenosides), may offer therapeutic potential in treating these infectious diseases. TCM may modulate immune responses by downregulating the expression of TRIM29 and TRIM18, thereby mitigating the inflammatory damage caused by these viral infections. These findings provide important clues for the development of novel herbal therapies based on the cGAS-STING pathway and open up new directions for immunomodulation in a variety of diseases.

Studying the targeting of the cGAS-STING pathway by TCM reflects the unique advantages of TCM in immunomodulation, providing both a scientific basis for modernizing traditional medicine and a new strategy for immunotherapy. However, while studies have demonstrated the potential of TCM in modulating the cGAS-STING pathway, more high-quality research is needed to validate these effects for true clinical applications. Meanwhile, an in-depth understanding of the mechanism of action of TCM can help promote the modernization of TCM and enhance its value for clinical application (204). In the future, with in-depth studies on the mechanisms of the cGAS-STING pathway, TCM may become an effective tool for modulating immune and inflammatory responses, bringing new hope for the treatment of a variety of diseases.
